# Supplementary material for: Analysis of miRNA Expression in Patients with Rheumatoid Arthritis during Olokizumab Treatment
Source: J Pers Med. 2020 Oct 31;10(4):205. doi: 10.3390/jpm10040205 (PMC7712090; doi:10.3390/jpm10040205)
Supplement: Supplementary file 1 [file jpm-10-00205-s001.pdf]

Supplementary 1. Statistically significant results of an association analysis between the baseline miRNA expression and the olokizumab therapy effectiveness

MiRNA	Baseline miRNA expression*	AUC (95% CI)	p-value	Cut-off point	Sensitivity (%)	Specificity (%)
			**ACR20. Week 12**			
**miR-29**		**0.633 (0.518, 0.747)**	**0.0229**	**-12.65**	**39.4**	**87.5**
Responders	-12.1538 ± 1.2822					
Non-responders	-11.6128 ± 1.3069					
**miR-451**		**0.644 (0.527, 0.762)**	**0.0160**	**-11.20**	**74.6**	**56.3**
Responders	-12.3176 ± 2.4865					
Non-responders	-11.3644 ± 2.0027					
			**ACR20. Week 24**			
**miR-29**		**0.667 (0.548, 0.787)**	**0.0061**	**-12.14**	**55.4**	**85.0**
Responders	-12.1329 ± 1.2758					
Non-responders	-11.3751 ± 1.2929					
			**ACR50. Week 12**			
**miR-26b**		**0.629 (0.520, 0.737)**	**0.0200**	**-13.92**	**68.8**	**56.4**
Responders	-14.2601 ± 1.2411					
Non-responders	-13.6917 ± 1.3413					
**miR-29**		**0.636 (0.528, 0.744)**	**0.0133**	**-12.73**	**43.8**	**83.6**
Responders	-12.3470 ± 1.1340					
Non-responders	-11.6704 ± 1.3764					
**miR-451**		**0.740 (0.643, 0.836)**	**<0.0001**	**-12.08**	**75.0**	**69.1**
Responders	-13.0743 ± 1.9622					
Non-responders	-11.1026 ± 2.3435					
**miR-522**		**0.656 (0.549, 0.762)**	**0.0042**	**-17.46**	**70.8**	**57.4**
Responders	-18.1829					

	± 2.0193					
Non-responders	-17.0616 ± 1.9689					
			**ACR50. Week 24**			
**miR-26b**		**0.614 (0.505, 0.724)**	**0.0403**	**-13.92**	**66.1**	**55.3**
Responders	-14.2157 ± 1.2008					
Non-responders	-13.6479 ± 1.4009					
**miR-29**		**0.634 (0.527, 0.741)**	**0.0144**	**-12.73**	**41.1**	**85.1**
Responders	-12.2898 ± 1.2052					
Non-responders	-11.6235 ± 1.3452					
			**DAS28. Week 12**			
**miR-26b**		**0.621 (0.510, 0.732)**	**0.0322**	**-14.53**	**50.0**	**77.0**
Responders	-14.2909 ± 1.2502					
Non-responders	-13.7264 ± 1.3280					
**miR-451**		**0.668 (0.562, 0.775)**	**0.0019**	**-12.25**	**69.0**	**67.2**
Responders	-12.8276 ± 2.0048					
Non-responders	-11.4664 ± 2.4708					
**miR-522**		**0.682 (0.577, 0.786)**	**0.0006**	**-17.83**	**69.0**	**63.3**
Responders	-18.3930 ± 1.7374					
Non-responders	-17.0266 ± 2.0959					
			**DAS28. Week 24**			
**miR-26b**		**0.703 (0.603, 0.804)**	**<0.0001**	**-13.92**	**73.1**	**60.8**
Responders	-14.4290 ± 1.0932					
Non-responders	-13.4749 ± 1.3670					
**miR-29**		**0.665 (0.560, 0.770)**	**0.0020**	**-11.64**	**82.7**	**47.1**
Responders	-12.3797					

	± 1.0621
Non-responders	-11.5841 ± 1.4188

*Data presented as mean ± SD.
SD – standard deviation; ACR20 (ACR50) - American College of Rheumatology 20% (50%) improvement response criteria; DAS28 – Disease Activity Score 28-joint Count; ROC - Receiver operating characteristic; AUC - Area under the ROC curve.
A cut-off point maximizes the sum of sensitivity and specificity, the corresponding sensitivity and specificity values, and the p-value for testing the null hypothesis that the AUC is equal to 0.5.
The relative miRNA expression (dCt) was determined as Ct of the reference RNA (cel-miR-39-3p) – Ct of the studied miRNA.

Supplementary 2. An association analysis between the baseline miRNA expression and olokizumab therapy effectiveness. Univariate and multivariate logistic regression.

	OR (95% CI)	p-value
**ACR20. Week 12**		
Univariate logistic regression		
Baseline miR-29	0.73 (0.52, 1.01)	0.0572
Baseline miR-451	0.84 (0.70, 1.01)	0.0654
Male gender	2.75 (0.57, 13.21)	0.2064
Age (years)	0.99 (0.95, 1.02)	0.4248
Weight (kg)	0.98 (0.96, 1.01)	0.2386
Disease duration (years)	1.03 (0.97, 1.10)	0.3302
Baseline DAS28	1.56 (0.81, 3.03)	0.1853
**ACR20. Week 24**		
Univariate logistic regression		
Baseline miR-29	0.65 (0.45, 0.95)	0.0249
Male gender	3.21 (0.39, 26.25)	0.2768
Age (years)	0.99 (0.95, 1.03)	0.5643
Weight (kg)	1.00 (0.97, 1.03)	0.9409
Disease duration (years)	1.00 (0.93, 1.07)	0.9668
Baseline DAS28	1.49 (0.68, 3.27)	0.3198
**ACR50. Week 12**		
Univariate logistic regression		
Baseline miR-26b	0.70 (0.51, 0.97)	0.0332
Baseline miR-29	0.64 (0.46, 0.91)	0.0116
Baseline miR-451	0.63 (0.50, 0.80)	0.0001
Baseline miR-522	0.74 (0.59, 0.93)	0.0082

Male gender	0.98 (0.31, 3.14)	0.9724
Age (years)	1.00 (0.97, 1.03)	0.9248
Weight (kg)	1.00 (0.97, 1.02)	0.9484
Disease duration (years)	1.04 (0.99, 1.10)	0.1479
Baseline DAS28	1.23 (0.68, 2.20)	0.4902
Multivariate logistic regression		
Baseline miR-451	0.63 (0.50, 0.80)	<0.0001
**ACR50. Week 24**		
Univariate logistic regression		
Baseline miR-26b	0.71 (0.51, 0.97)	0.0342
Baseline miR-29	0.65 (0.47, 0.91)	0.0131
Male gender	3.19 (0.82, 12.36)	0.0935
Age (years)	0.99 (0.96, 1.02)	0.4875
Weight (kg)	0.99 (0.97, 1.02)	0.6243
Disease duration (years)	1.04 (0.98, 1.10)	0.1726
Baseline DAS28	1.24 (0.69, 2.24)	0.4679
Multivariate logistic regression		
Baseline miR-29	0.65 (0.47, 0.91)	0.0131
**DAS28. Week 12**		
Univariate logistic regression		
Baseline miR-26b	0.70 (0.50, 0.98)	0.0365
Baseline miR-451	0.76 (0.62, 0.92)	0.0059
Baseline miR-522	0.68 (0.53, 0.86)	0.0017
Male gender	1.83 (0.57, 5.91)	0.3099
Age (years)	0.99 (0.96, 1.02)	0.4729
Weight (kg)	0.99 (0.96, 1.01)	0.3947

Disease duration (years)	1.03 (0.97, 1.09)	0.3014
Baseline DAS28	0.70 (0.38, 1.28)	0.2414
Multivariate logistic regression		
Baseline miR-522	0.68 (0.53, 0.86)	0.0017
**DAS28. Week 24**		
Univariate logistic regression		
Baseline miR-26b	0.51 (0.35, 0.75)	0.0006
Baseline miR-29	0.59 (0.41, 0.84)	0.0034
Male gender	3.81 (0.98, 14.77)	0.0530
Age (years)	1.00 (0.97, 1.03)	0.9948
Weight (kg)	1.00 (0.98, 1.03)	0.9159
Disease duration (years)	0.96 (0.91, 1.02)	0.2018
Baseline DAS28	0.73 (0.40, 1.31)	0.2921
Multivariate logistic regression		
Baseline miR-26b	0.50 (0.33, 0.74)	0.0005
Male gender	4.44 (1.05, 18.72)	0.0423

ДИ = Доверительный интервал; ОШ = Отношение шансов; СРБ = С-реактивный белок; ACR20 = Улучшение состояния на 20% согласно критериям Американской коллегии ревматологов; ROC = Receiver operating characteristic, рабочая характеристика приёмника.
В однофакторную модель включались следующие факторы: пол, возраст, Weight (kg), Disease duration (years), исходная активность заболевания (DAS28-СРБ).
В многофакторную модель включались факторы, значимые на уровне 0.1 в однофакторном анализе, с помощью stepwise forward logistic regression.
Относительный уровень экспрессии микроРНК (dCt) определялся по значению пороговых циклов (Ct) как Ct референсной РНК (cel-miR-39-3p) – Ct исследуемой микроРНК.
